# Insight to aspirin sorption behavior on carbon nanotubes from aqueous solution: Thermodynamics, kinetics, influence of functionalization and solution parameters

**DOI:** 10.1038/s41598-019-49331-6

**Published:** 2019-09-05

**Authors:** Mohamed R. Elamin, Babiker Y. Abdulkhair, Amin O. Elzupir

**Affiliations:** 1 Imam Mohammad Ibn Saud Islamic University (IMSIU), College of Science, Chemistry Department, Riyadh, Saudi Arabia; 2 Industrial research and consultancy center (IRCC), Khartoum North, Sudan; 3 Imam Mohammad Ibn Saud Islamic University (IMSIU), College of Science, Committee on Radiation and Environmental Pollution Protection, Riyadh, Saudi Arabia; 4 Sudan University of Science and Technology (SUST), College of Science, Chemistry Department, Khartoum, Sudan

**Keywords:** Pollution remediation, Carbon nanotubes and fullerenes

## Abstract

The chronic exposure to the pharmaceuticals and personal care products contaminants in water represent a serious public health problem to man and animal. We studied the removal of aspirin (Asp) as an example to these hazardous materials from an aqueous solution using functionalized (FMCNT) and pristine multiwall carbon nanotubes (PMCNT). The characterization of synthetic sorbents was examined with scanning electron energy-dispersive microscopy and transmission electron microscopy. The effects of adsorption time, sorbent mass, solution pH, ionic strength, and temperature were optimized. The functionalization increased the surface area from 151 to 181 m^2^ g^−1^. Consequently, the adsorption capacity increased from 41 mg g^−1^ to 58 mg g^−1^ for PMCNT and FMCNT, respectively. The results showed that the adsorption kinetic follows the pseudo-second-order model with very good agreement. Whereas, the adsorption mechanism study showed a partial agreement with the liquid-film diffusion model on PMCNT and FMCNT at 25 °C and 35 °C, respectively, with acceptable linear regression coefficients. The adsorption isotherm results revealed that the adsorption fits the Freundlich model. The thermodynamic study revealed that, Asp adsorption on both sorbents is exothermic, spontaneous and favorable. FMCNT showed relatively high removal efficiency when compared with the PMCNT when used for most of the conditions investigated.

## Introduction

The treatment of wastewater from organic and pharmaceutical contaminants is a major challenge for many countries around the world, due to the increasing need for safer water for agricultural and industrial operations. The contamination of water by these pollutants poses a real danger to the environment, as well as to human and animal health. The presence of pharmaceuticals in urban and wastewater results from the secretion of these compounds in urine and feces after being partially metabolized by man and animal. Different operations in the pharmaceutical process, such as washing of solid cake in the machine and equipment^1^, significantly contribute to this issue. Also, these pollutants can enter the water cycle from illegal discarding of the expired materials in the sewage system.

Pharmaceuticals and personal care products (PPCPs) have been classified as a group of emerging anthropogenic contaminants, distinguished by their bioactivity and high solubility. PPCPs contain different groups of human and animal pharmaceutical products that are found in low concentrations in municipal and/or industrial wastewater. These products may cause serious public health problems through chronic exposure^2–4^. Although Urban Wastewater Treatment Plants have set standards for many pollutants, such as chemical oxygen demand, biological oxygen demand, and suspended solids, the threshold limits for many PPCPs were not fully declared. This may indicate that the presence of these PPCPs in wastewater at any concentration is unacceptable^5^.

A review of the literature indicates that there are many methods studied to remove PPCPs from wastewater, such as oxidation^6,7^, membrane bioreactors^8^, ZnO-sepiolite heterostructured materials and solar photocatalytic degradation^9^, and electrocoagulation^10^. Among these sorbents, multiwall carbon nanotubes (MCNTs) have been investigated as a pure material and as composites with other chemicals, and they were found to be powerful and cheap methods for the removal of PPCPs from wastewater^11–16^.

Aspirin is one of the most widely produced and consumed drugs, and is therefore one of the most persistent pharmaceutical pollutants in water resources and wastewater^17^. In the present study, the PMCNT and FMCNT were synthesized and characterized, in order to be used as sorbent for the removal of Asp from water as an example of PPCPs. The conditions of the adsorption such as the influence of functionalization, sorbent mass, adsorption time, solution pH, ionic strength, and temperature were investigated and optimized. The adsorption isotherms were also studied and discussed.

## Results and Discussion

### Characterization of carbon nanotubes

**Figure 1:**
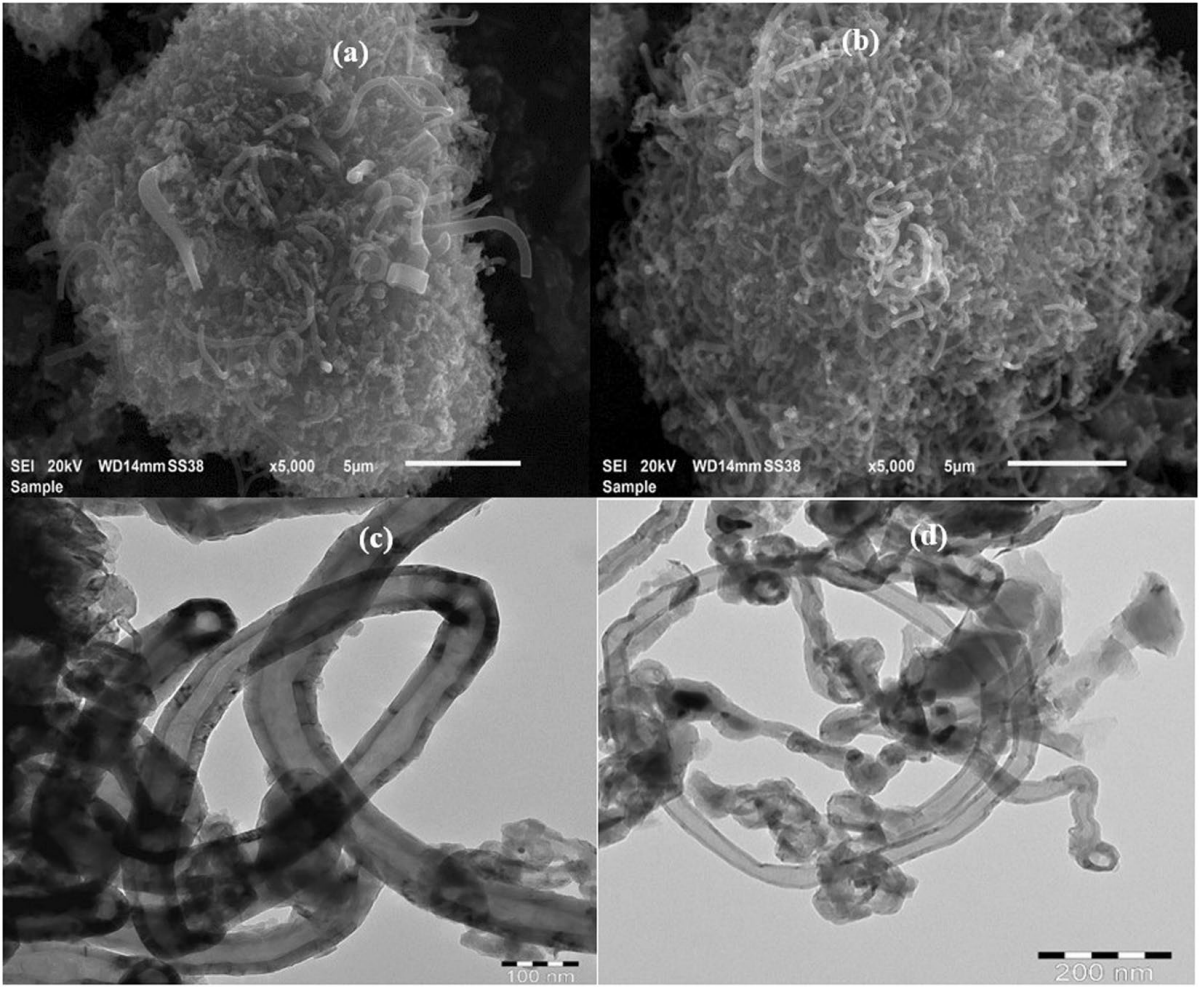
SEM images for (**a**) PMCNTs, (**b**) FMCNTs, and TEM images of (**c**) PMCNTs, (**d**) FMCNTs.

The synthesis of carbon nanotubes on cheap and simple substrates without using an external catalyst is an attractive option. The elimination of transition metal catalysts produces relatively pure carbon nanotubes (PMCNTs) that are free of metal oxide residues^18^. In this study, 6.0 g of PMCNTs were collected from the inner wall of a stainless-steel cylinder in chemical vapor deposition experiments. Acid treatments of the PMCNTs remove traces of the steel cylinder metals and oxidize the surface of FMCNT, introducing different functional groups such as hydroxyl, carbonyl, and carboxylic acid groups via the action of nitric acid^19^. The morphology of the two samples was investigated using a scanning electron microscope. SEM images of the PMCNTs showed a lump of nested nanotubes, as well as some nanofibers with a large diameter and semi-cylindrical shape. Residues of amorphous carbon appeared to accompany the pristine nanotubes, as seen in Fig. 1a. SEM images of FMCNTs (Fig. 1b) showed relatively pure and clear CNTs, indicating the success of the acid purification step in the removal of interfering carbonaceous materials.

**Figure 2:**
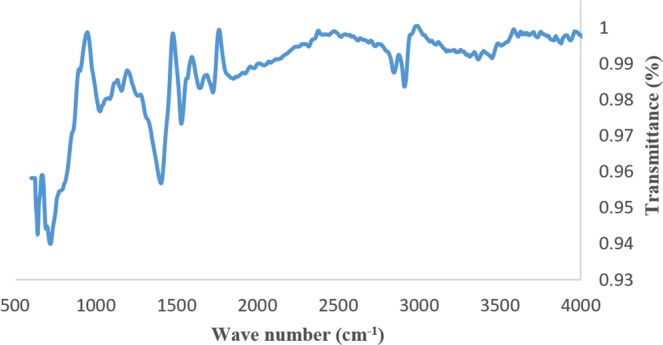
FT-IR chromatogram for the functionalized carbon nanotubes.

The elemental composition of the two CNTs was estimated using EDX. The analysis revealed higher carbon content in the FMCNTs (~98.0%), while that of the PMCNTs was ~85.0%. Acid treatment of the PMCNTs reduced the steel cylinder element content in the PMCNTs to very low values and freed the FMCNTs from the carbonaceous materials. The functionalization was confirmed by FT-IR chromatogram (Fig. 2) and the results of Boehm titration^20^ showed that, the acid sites of FMCNTs were 251.2 μmole g^−1^. The content of interfering elements was found to be higher in the PMCNTs, as expected; in fact, the high value of the oxygen content in the crude PMCNTs (7.5%) can be attributed to the ease of oxidation of graphite and amorphous carbon when compared with the difficult oxidation of CNTs.

### TEM characterization of PMCNTs and FMCNTs

TEM images of PMCNTs and FMCNTs confirmed the synthesis of CNTs on the stainless-steel substrate. Figure 1c shows the presence of a long-coiled CNTs accompanied by black carbonaceous materials. Figure 1d shows relatively pure FMCNTs, which resulted from the purification and oxidation step. SEM and TEM images showed variable nanotube diameters due to the different sizes of the steel nanoparticles, which served as growth roots for the PMCNTs on the stainless-steel cylinder. The amorphous carbon and the graphitic materials were found to be attached to the surface of the thick walls of both PMCNTs and FMCNTs, and they were even embedded between the concentric, thick, multi-walls tubes.

### Nitrogen adsorption/desorption isotherms

**Figure 3:**
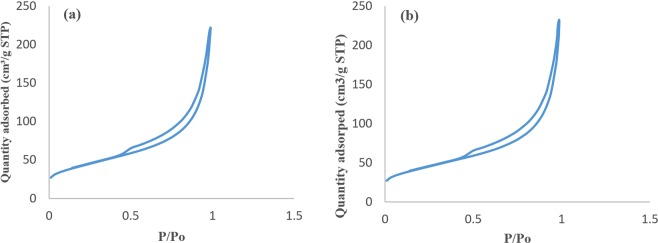
Nitrogen adsorption/desorption isotherms (**a**) for PMCNTs and (**b**) FMCNTs.

According to the original IUPAC classification, the isotherm obtained for the prepared PMCNTs and FMCNTs exhibit an H3 type hysteresis loop (Fig. 3), which corresponds to typical mesoporous structure. The BET Surface Area of PMCNT and FMCNT was found to be 151 and 181 m^2^ g^−1^, the dimeter (BJH) was 8.43 and 11.35 nm, and the total volume was 1.34 and 2.2 cm^3^ g^−1^ respectively^2^. The obtained results indicate that, the properties of the prepared CNTs was improved due to purification and functionalization.

### Adsorption studies

**Figure 4:**
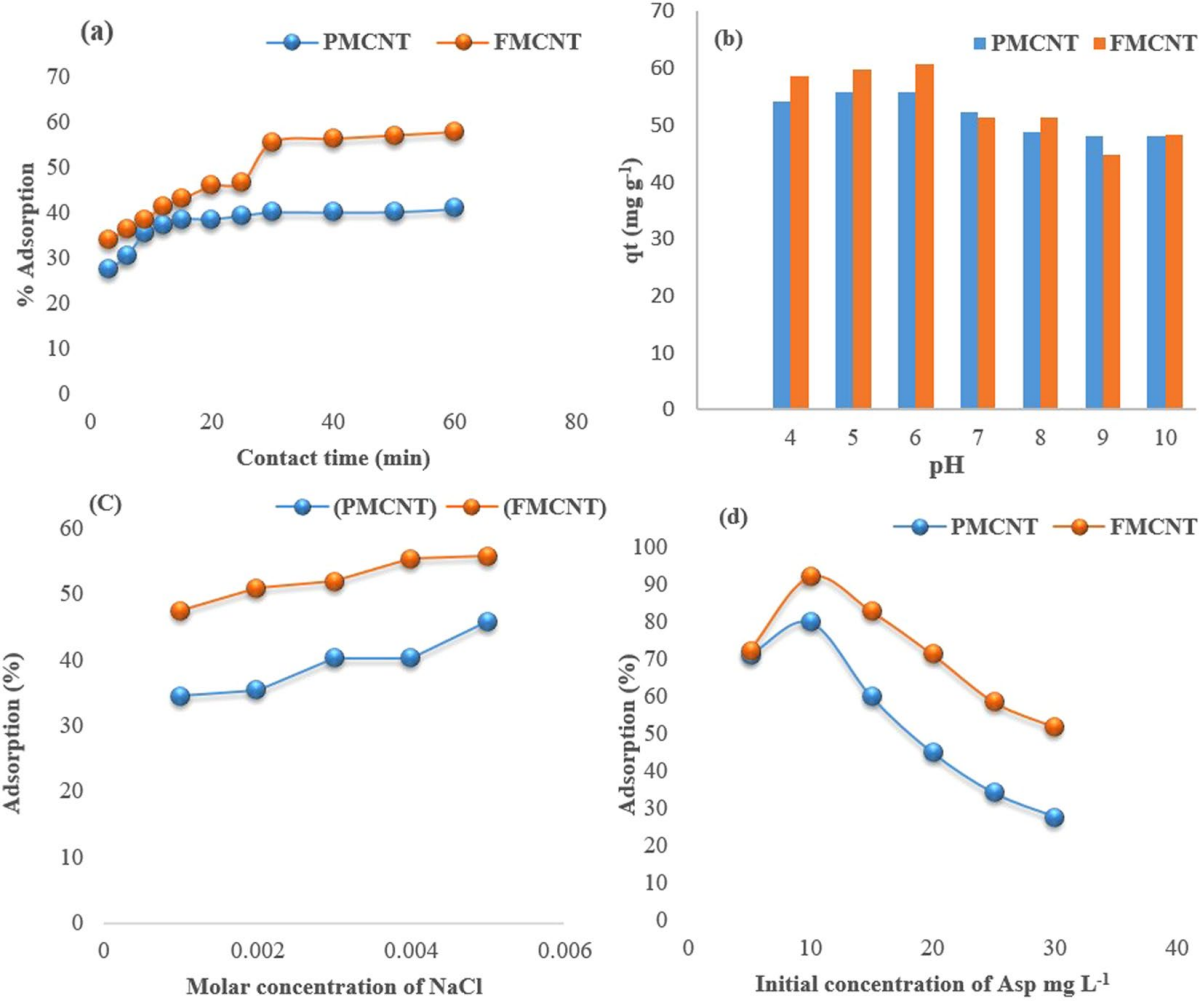
The effect of (**a**) contact time, (**b**) pH, (**c**) the ionic strength, and (**d**) the initial concentration on the adsorption of Asp by PMCNTs and FMCNTs from aqueous solutions at room temperature.

The adsorption of Asp served as an example of a pharmaceutical pollutant obtained from aqueous solutions by carbon-based adsorbents and controlled by Van der Waals interactions. To improve the absorptivity properties of carbon, PMCNTs and FMCNTs were synthesized. Both PMCNTs and FMCNTs were used for the removal of Asp from an aqueous solution. The factors that may affect adsorption, such as adsorbent mass, the initial pollutant concentration, contact time, pH level, ionic strength, and temperature, were investigated. The effect of PMCNT and FMCNT mass on the adsorption profile of Asp was studied. Generally, it was observed that the percentage of Asp adsorption increased in association with increases in CNTs mass. This can be attributed to the increase of adsorption sites, which resulted from increasing the CNTs mass to interact with the Asp molecules. The effect of contact time on the adsorption percentage of Asp by CNTs was demonstrated in Fig. 4a. The figure shows that the percentage adsorption of Asp increased over time for the first 30 minutes, after which no further improvement in adsorption percentage was observed; thus, equilibrium was achieved for both sorbents. Given the high availability of active binding sites on the CNTs surface during the first quarter of testing time, it was clear that the CNTs could rapidly and efficiently remove PPPCs from aqueous solutions. Also, the figure shows that the efficiency of FMCNTs was slightly higher than that for the corresponding PMCNTs.

The effect of pH on the adsorption of Asp by PMCNT and FMCNT at pH values ranging from 3.0–10.0 was studied. The results are presented in Fig. 4b. In general, the maximum adsorption capacity was found at pH 6 for both sorbents. The relatively different adsorption percentages might indicate that adsorption is dependent on both the sorbate and sorbents. The decrease in adsorption percent at high pH levels of 9 and 10 can be attributed to the deprotonation of Asp (Asp pKa = 3.57), and thus the electrostatic repulsion between the negatively charged Asp and CNTs. Accordingly, CNTs may be recycled by strong alkaline solutions. Furthermore, these results highlight the important role of pH in the removal of PPCPs.

The ionic strength was known to affect the electrostatic and hydrophobic interactions in a solution mixture containing an adsorbate and a sorbent. Herein, the effect of ionic strength was investigated and the results are presented in Fig. 4c. The figure shows that no significant effects were observed after increasing ion strength. This trend indicates that the CNTs can remove the PPCPs without significant effect from the soluble ionic salt frequently present in water.

The effect of the initial concentrations of Asp on adsorption efficiency was investigated and the results are shown in Fig. 4d. The figure indicated that the adsorption efficiency decreased upon increasing the initial concentrations of both sorbents. This is not an unexpected result given the saturation of CNT adsorption sites after reaching concentrations of 10 mg L^−1^. However, the efficiency of FMCNTs was a little bit higher than that for the corresponding PMCNTs. This may be due to the abundance of different functional groups in the outer walls of the FMCNTs.

**Figure 5:**
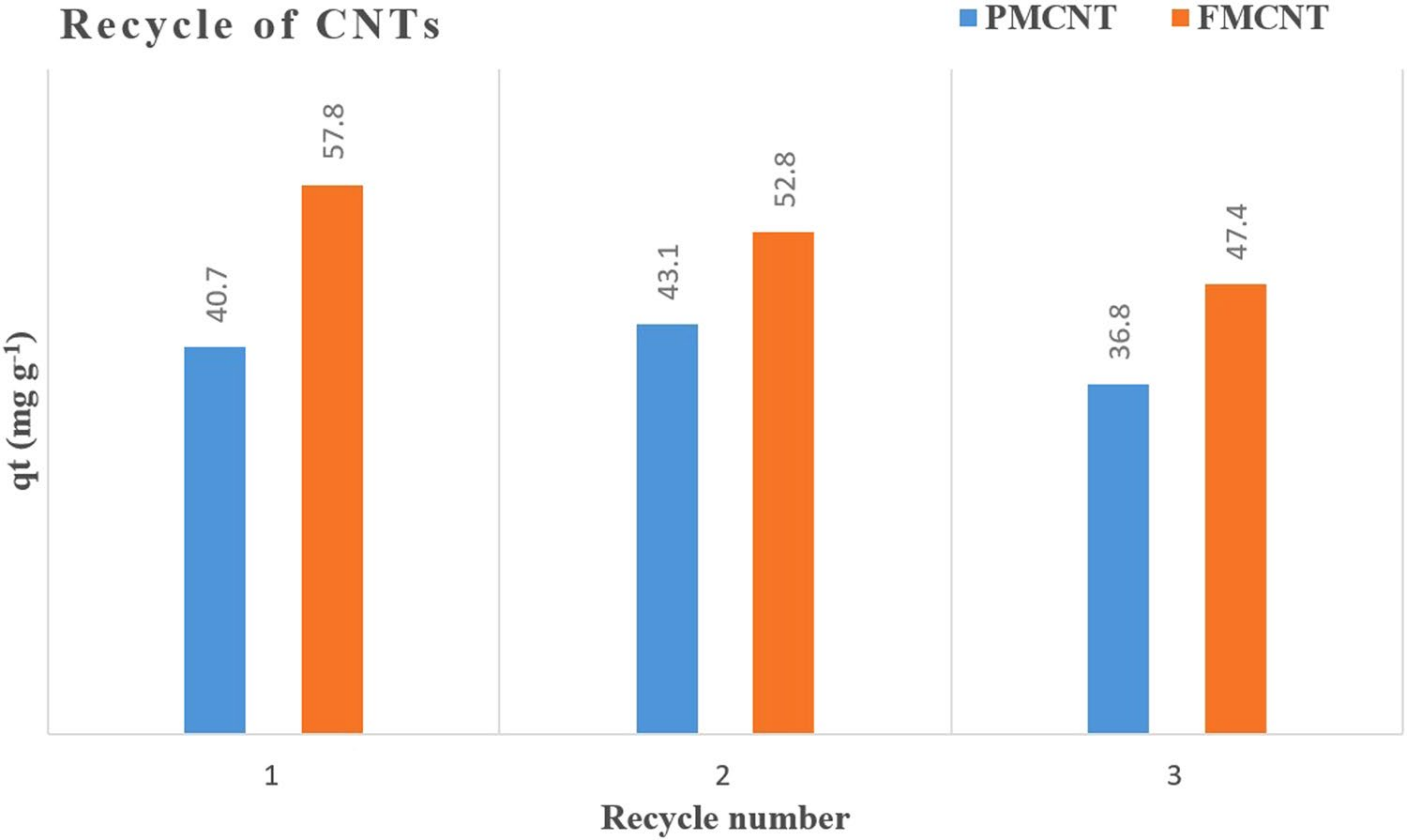
Adsorption efficacy of the recycled CNTs for the removal of aspirin after each retrieval.

In order to conserve the ratio of sorbent: aspirin, the volume of aspirin standard was retained even after two cycles of sorption/desorption process, indicating its good reusability. Regeneration efficiency for each cycle was monitored as adsorption percentage as shown in Fig. 5. However, the efficiency of FMCNT was slightly reduced after two cycles, in contrast the first recycle of PMCNT observed more efficient than the crude PMCNT, this can be attributed to pores opening by ethanol used for regeneration.

### Adsorption kinetics

The effects of temperature on the adsorption of Asp on PMCNTs and FMCNTs in an aqueous solution were studied. It was found that, raising the solution temperature decreases the adsorption capacity of Asp in both PMCNT and FMCNT sorbents. The adsorption capacity of Asp on FMCNT decreased from 58 mg g^−1^ to 23 mg g^−1^ and 22 mg g^−1^ at 25 °C, 35 °C, and 45 °C, respectively. On the other hand, the adsorption capacity of Asp on PMCNT decreased from 41 mg g^−1^ to 23 mg g^−1^ and 21 mg g^−1^ at 25 °C, 35 °C, and 45 °C, respectively. The reduction in adsorption capacity of Asp in both CNTs sorbents caused by increasing the solution temperature highlights how the adsorption process is exothermic.

The effect of solution temperature on the adsorption rate and mechanisms were also investigated using various kinetic models. These models include pseudo-first-order and pseudo-second-order kinetic models using Eqs (1) and (2), as based on the Lagrange equation^21^:

1 $$\mathrm{ln}({q}_{e}-{q}_{t})=\,\mathrm{ln}\,{q}_{e}-{{\rm{k}}}_{1}t$$

2 $$\frac{{\rm{t}}}{{q}_{t}}=\frac{1}{{{\rm{k}}}_{2}{q}_{e}^{2}}+\frac{{\rm{t}}}{{q}_{e}}$$

where k_1_ (min^−1^) and k_2_ (g (mg min)^−1^) are the adsorption rate coefficients of the pseudo-first-order and the pseudo-second-order models, respectively.

Moreover, q_t_ and q_e_ are the adsorbed amount per unit mass at time t and at equilibrium, respectively.

Table 1 shows the experimental and calculated parameters of the kinetic models. The adsorption of Asp on both sorbents (FMNCTs and PMCNTs) at different temperatures did not have a linear effect. It was also observed that the q_e_ (calculated) was far from the experimental value q_e_ (experimental); thus, the adsorption process does not follow a pseudo-first-order model. In contrast, applying the pseudo-second-order rate model at different temperatures produced a linear relation with an excellent correlation coefficient (*R*^2^ ≥ 0.997) for both sorbents, and it was also discovered that the q_e_ (calculated) was in good agreement with experimental value q_e_ (experimental). These results reflect the notion that the adsorption process follows the pseudo-second-order kinetic model.

**Table 1:** The adsorption kinetic parameters and thermodynamic parameters at pH 6.0 and different temperatures for Asp adsorption on PMCNT and FMCNT sorbents.

PMCNT					FMCNT				
Pseudo-first-order kinetic model									
Temperature °C	q_e_ exp. (mg/g)	q_e_ cal. (mg/g)	k_1_	R^2^	Temperature °C	q_e_ exp. (mg/g)	q_e_ cal. (mg/g)	k_1_	*R* ^2^
25	33.25	0.92	0.0135	0.2292	25	36.92	14.70	0.1083	0.4275
35	23.24	2.09	0.0743	0.6251	35	23.52	6.13	1.0489	0.6595
45	22.70	0.17	0.0068	0.0037	45	23.70	3.04	0.0747	0.4263
**Pseudo-second-order kinetic model**									
**Temperature °C**	**q**_**e**_ **exp. (mg/g)**	**q**_**e**_ **cal. (mg/g)**	**K**_**2**_	**R** ^**2**^	**Temperature °C**	**q**_**e**_ **exp. (mg/g)**	**q**_**e**_ **cal. (mg/g)**	**K**_**2**_	***R*** ^**2**^
25	33.25	31.65	0.0399	0.9975	25	36.92	38.91	0.0085	0.9994
35	23.24	23.31	0.1017	0.9998	35	23.52	23.75	0.0477	0.9997
45	22.70	22.12	0.1081	0.9997	45	23.70	22.52	0.0861	0.9968
**Intra-particle diffusion model**									
**Temperature °C**	**K**_**id**_ **(mg/g min** ^**1/2**^ **)**		**C (mg/g)**	***R*** ^**2**^	**Temperature °C**	**K**_**id**_ **(mg/g min** ^**1/2**^ **)**		**C (mg/g)**	***R*** ^**2**^
25	2.1905		17.006	0.6664	25	2.3501		20.223	0.7904
35	0.0976		22.4	0.5675	35	0.179		22.018	0.878
45	0.0425		21.958	0.0201	45	0.1998		20.594	0.4776
**Liquid film diffusion model**									
**Temperature °C**	**K**_**fd**_ **(min** ^**−1**^ **)**		***R*** ^**2**^		**Temperature °C**	**K**_**fd**_ **(min** ^**−1**^ **)**		***R*** ^**2**^	
25	0.0304		0.9608		25	0.124		0.3159	
35	0.0008		0.5645		35	0.0012		0.9313	
45	0.0013		0.3762		45	0.0003		0.0360	

### Adsorption rate-controlling mechanism

Adsorption is a multi-step process that involves the transport of Asp from the aqueous solution to the surface of the PMCNT and FMCNT particles, followed by the diffusion of sorbate molecules through the CNT boundary layer to the external surface; this step is followed by adsorption at the active sites of CNTs. The last step is intra-particle diffusion through pores and aggregates.

Intra-particle diffusion and the liquid film-diffusion kinetic models were investigated at different temperatures using Eqs (3) and (4), respectively:

3 $${q}_{t}={k}_{id}t+C$$

where q_t_ is adsorption capacity at time (t), k_id_ (mg/g min^1/2^) is the rate constant for intra-particle diffusion, and C (mg/g) is a constant proportional to the boundary layer thickness^2^.

4 $$\mathrm{ln}(1-F)=-\,{k}_{fd}\ast t$$

where F is the equilibrium fractional attainment, while k_fd_ (min^−1^) represents the film diffusion rate coefficient^2^.

If the adsorption process is controlled by intra-particle diffusion, the plot of q_t_ against the square root of time yields a straight line that passes through the point of origin. The same holds true for adsorption, which is controlled by a liquid film-diffusion kinetic model; however, herein, the plot is of ln (1–F) against the contact time of adsorption on CNTs.

**Figure 6:**
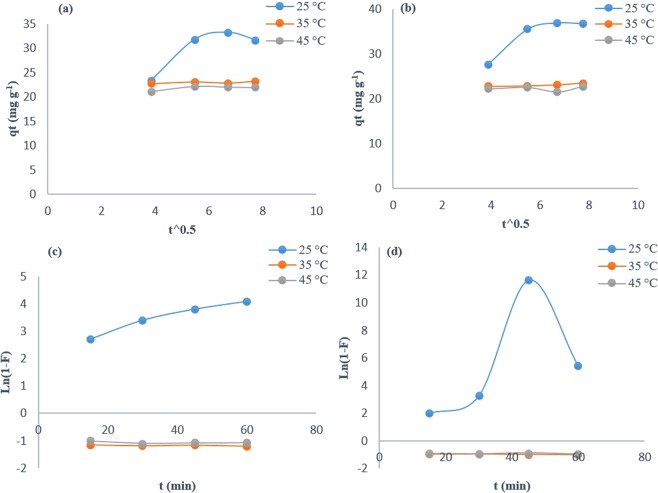
The intra-particle diffusion (**a,b**) and the liquid film-diffusion (**c,d**) kinetic models for Asp adsorption on PMCNT (**a,c**) and FMCNT (**b,d**) sorbents at pH 6.0.

The results of the investigated kinetic models for the rate-controlling mechanism of Asp adsorption on CNTs is presented in Fig. 6.

It is obvious that the adsorption mechanism of Asp partially fits the liquid film-diffusion model on PMCNTs at 25 °C and on FMCNTs at 35 °C, with acceptable linear regression coefficients, as shown in Table 1. These results suggest that the adsorption mechanism at the stated conditions is not controlled by the intra-particle diffusion during the entire adsorption time for both sorbents.

According to the above results, the best adsorption capacities of Asp by PMCNTs and FMCNTs at 25 °C were 41 mg g^−1^ and 58 mg g^−1^, respectively. These results were compared with other results of Asp removal from the literature as shown in Table 2. It is obvious that the adsorption capacities reported in this study are higher than the majority of the reported values. Also, the time required to reach equilibrium in our study is lower than in many other studies. This results exhibited the efficiency of CNTs for the removal of Asp from aqueous solution.

**Table 2:** The aspirin adsorption with prepared CNTs compared with the literature results of many other adsorbents.

Sorbent	Temperature (Kelvin)	Contact time (min)	Adsorption capacity (mg g^−1^)	Reference
PMCNT	298	15	41	This study
FMCNT	298	30	58	This study
Graphene nanoplatelets	296	10	12.98	^2^
Natural zeolites and clays	303	120	3.36	^22^
Molecularly imprinted polymers	298	120	41.4	^23^
Molecularly imprinted polymers	298	720	9.72	^24^
Tyre Waste	303	60	40.4	^17^

### Adsorption isotherm studies

The Freundlich and Langmuir models were employed. Equations 5 and 6 represent the linearized equations for Freundlich and Langmuir, respectively:

5 $$\mathrm{ln}\,{q}_{e}=\,\mathrm{ln}\,k+\frac{1}{n}\,\mathrm{ln}\,{c}_{e}$$

6 $${q}_{e}=(\frac{{K}_{l}b{C}_{e}}{1+b{C}_{e}\,})$$

**Figure 7:**
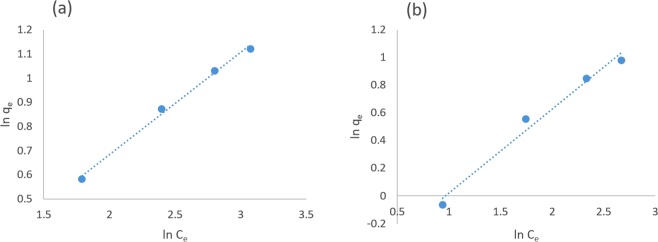
The Freundlich adsorption isotherm for Asp on (**a**) PMCNT and (**b**) FMCNT sorbents at 25 °C and pH 6.0.

The Asp adsorption isotherm on both sorbents followed the linearized Freundlich model (Fig. 7). In contrast, the adsorption isotherm did not align with the Langmuir model for both sorbents using the same experimental conditions. The parameters of adsorption isotherms for both models are reported in Table 3.

**Table 3:** The isotherms parameters, thermodynamic parameters, and adsorption percentage of Asp from real samples on PMCNT and FMCNT at pH 6.0.

Isotherms parameters						
Freundlich						
Parameters	K (l/g)		1/n		R^2^	
PMCNTs	0.851		0.423		0.994	
FMCNTs	0.557		0.607		0.981	
**Langmuir**						
**Parameters**	**b (l/g)**		**K**_**L**_ **(mg/g)**		***R*** ^2^	
PMCNTs	−1.817		8.278		0.652	
FMCNTs	0.746		21.552		0.975	
**Thermodynamic parameters**						
**PMCNTs**						
**Asp conc.(mg L** ^**−1**^ **)**	**ΔH**°	**ΔS**°	**ΔG**° **(298 K)**	**ΔG**° **(308 K)**		**ΔG**° **(318 K)**
5	−47.239	−0.144	−4.305	−2.864		−1.423
10	−42.598	−0.131	−3.446	−2.132		−0.818
15	−28.863	−0.093	−1.371	−0.108		−0.095
**FMCNTs**						
**Asp conc.(mg L** ^**−1**^ **)**	**ΔH**°	**ΔS**°	**ΔG**° **(298 K)**	**ΔG**° **(308 K)**		**ΔG**° **(318 K)**
5	−83.178	−0.255	−7.112	−4.561		−2.007
10	−70.864	−0.217	−6.196	−4.026		−1.856
15	−49.821	−0.154	−3.983	−2.444		−0.906
**Adsorption percentage of Asp by FMCNTs and PMCNTs from environmental samples**						
	**PMCNT**			**FMCNT**		
**Asp conc.(mg L** ^**−1**^ **)**	**Tw**	**AGw**	**Ww**	**Tw**	**AGw**	**Ww**
1	65.5	60.7	36.9	76.3	70.1	43.5
5	92.5	84.2	59.5	95.2	85.9	62.0
10	85.7	80.3	64.2	89.3	81.0	69.0

### Thermodynamic study

The enthalpy (Δ*H*◦), entropy (Δ*S*◦) and free energy (Δ*G*◦) can be evaluated by the following Eqs (7–9):

7 $$Kc=\frac{{C}_{Ae}}{{C}_{e}}$$

8 $$ln\,Kc=\frac{-\varDelta {H}^{o}}{RT}+\frac{\varDelta {S}^{o}}{R}$$

9 $$\Delta {G}^{o}=\Delta {H}^{o}-T\Delta {S}^{o}$$

where *K*c is the equilibrium constant, *C*_Ae_ and *C*_e_ represents the adsorbed and the remained Asp concentration, respectively.

The thermodynamic parameters were calculated on a temperature range of 298–318 Kelvin as shown in Table 3. The negative Δ*G*◦ values reflected that, the adsorption process is spontaneous and less negative value with increase of temperature shows that an increase in temperature disfavors the sorption process. The negative Δ*H*◦ values indicated that the adsorption process is exothermic and the negative values of Δ*S*◦ indicated that, the adsorption of Asp on FMCNT is more favorable than on PMCNT.

### Application to environmental water samples

The obtained results (Table 3) confirmed the applicability of PMCNTs and FMCNTs for the removal of Asp environmental water samples. The Highest adsorption percentage of Asp was obtained with the Tw followed by AGw and Tw samples. The lowest adsorption percentage in Ww sample may be attributed to the competition of its constituents such as salts and organic compounds with the Asp on sorbents sites. Also, the high concentration of alkali and alkaline earth salts in the AGw sample may explain the low removal of Asp in AGw when compared with Tw sample.

## Conclusion

The removal of Asp as an example of PPCPs from aqueous solutions by PMCNTs and FMCNTs was investigated. The present study shows that the CNTs are effective adsorbents for Asp removal. From the kinetics investigations, it was observed that the adsorption of Asp was rapid, as it approached equilibrium over a short period of time (30 min). The adsorption capacities were found to be 41 mg g^−1^ and 58 mg g^−1^ on PMCNTs and FMCNTs, respectively. The experimental data related to adsorption isotherms were in good agreement with Freundlich model, and they obeyed pseudo-second-order processes with good linearity. The data were also partially in alignment with the liquid film-diffusion model for PMCNTs at 25 °C and for FMCNTs at 35 °C, with acceptable linear regression coefficients. Finally, we can conclude that PMCNTs and FMCNTs can remove PPCPs contaminants from water sources very efficiently. The real challenge remains in the process of developing and manufacturing CNTs commercially and at a lower cost.

## Materials and Methods

### Synthesis of multiwall CNTs

Multiwall CNTs were prepared using a chemical vapor deposition (CVD) method on the inner surface of a commercial stainless-steel cylinder used as a reaction chamber in a tubular heat-controlled muffle furnace set at 700 °C (Nabertherm GmbH, Lilienthal, Germany). The tube was 80 cm long and 4 cm in diameter. Then, 80 cm^3^ of pure ethanol (Sigma Aldrich Co., St Louis, MO, USA) was boiled on a hot plate and a current of nitrogen gas (40 dm^3^/hour) was used to introduce the ethanol vapor to the reaction chamber at 700 °C. All the ethanol vapor was introduced to the chamber in 20 minutes. The reactor was then allowed to cool under a nitrogen atmosphere. The black soot was scratched from the tube using a plastic brush; it was weighed and preserved as a pristine carbon nanotube. Three grams of this soot were purified and functionalized by boiling the mixture in a 3:1 concentrated hydrochloric and nitric acid for 1 hour. The mixture was cooled and filtered on filter paper using a Buchner apparatus, washed until it reached a neutral pH, and dried at 80 °C in a drying oven for 6 hours. The functionalized soot was collected, ground in a porcelain mortar, and preserved as functionalized carbon nanotubes.

### Characterization of multiwall CNTs

The composition and morphology of pristine functionalized multiwall carbon nanotubes (PMCNTs) and (FMCNTs) were studied using a scanning electron–energy-dispersive X-ray technique (SEM-EDX; model JSM-IT300). The elemental composition was obtained by the energy-dispersive X-ray technique (EDX). The morphology of the two CNTs was characterized by scanning electron microscopy (SEM). PMCNTs and FMCNTs were imaged using transmission electron microscopy (TEM; model JEOL-1011); the results are shown in Fig. 1c,d.

### Analytical method

Aspirin (Asp) concentrations were measured using an ultraviolet (UV)-visible (VIS) spectrophotometer (model: double-beam spectro UV-VIS, UV D3500 LABOMED). The samples were prepared by dissolving a certain amount of Asp standard in a certain volume of deionized water to yield the desired concentrations. A series of concentrations was prepared, which ranged from 5–250 ppm. Absorbance was measured at a maximum lambda of 290 nm. The linear regression (*r*^2^) of our curve was found to be 0.9998. The un-adsorbed concentration was determined by withdrawing a certain portion of the mixture using a 5 mL syringe and filtered using a 0.45 µm nylon membrane syringe filter. The adsorbed Asp concentrations were calculated using the linear regression equation. Statistical analysis was performed using Microsoft excel (version 2016).

### Adsorption experiments

Some adsorption parameters, including adsorption time, adsorbent mass, solution pH, and initial Asp concentration, were investigated using batch experiments. For the pH experiment, the ionic strength in each buffered solution was determined and adjusted by sodium chloride (0.01 M) to have an electrical conductivity of 320 µS.

For adsorption time, an Asp solution containing 100.0 mg L^−1^ was prepared. The pH of the solution was adjusted to 6 using 0.1 M of HCl and 0.1 M of NaOH. Of this solution, 25 mL and 25 mg of PMCNTs or 25 mg of FMCNTs were placed into 50 mL plastic tubes. The mixture was shaken continuously for certain periods of time (3, 6, 9, 12, 15, 20, 25, 30, 40, 50, and 60 minutes). After these preset times had passed, the solution was immediately filtered through a nylon syringe filter (0.45 µm). A UV-VIS spectrophotometer was applied for absorbance measurement.

The percentage adsorption and adsorption capacity of Asp on PMCNT and FMCNT in aqueous solution was calculated using Eqs (7) and (8), as follows:

10 $$ \% \,Adsorption=\frac{({C}_{o}-{C}_{t})}{{C}_{o}}\ast 100 \% $$

11 $${q}_{t}=\frac{({C}_{o}-{C}_{t})\,V}{M},$$

where C_o_ is the initial concentration (mg L^−1^), C_t_ is the remaining concentration after a certain period of time (mg/L), q_t_ is the amount of Asp adsorbed by the PMCNTs and FMCNTs (mg/g), M is the PMCNT or FMCNT mass (g), and V is solution volume (L).

Each experiment was carried out in triplicate and the average was reported. Blank experiments were performed to evaluate the effect of glassware and nylon filter on the adsorption of Asp, and the effects were found to be negligible.

### Application to environmental water samples

Three samples were employed to explore the applicability of PMCNT and FMCNTs for the removal of Asp. The laboratory tap was opened for 15 minutes, after which 2.5 L tap water sample was collect by taking 250 ml every 5 minutes. The Arabic Gulf water sample (AGw) was collected from Al Khobar City - Saudi Arabia, while the waste water sample was collected from the Sewage Treatment Plant (Riyadh - Saudi Arabia). All samples were filtered through 0.22 nylon membrane filter preserved in a polypropylene bottle at 5 °C in the dark and used within 24 h. A samples of tap water (Tw), Arabic Gulf water (AGw), and wastewater (Ww), were filtered and used to explore the applicability of PMCNTs and FMCNTs to the removal of Asp from an environmental samples. Each sample was spiked to obtain 1, 5 and 20 mg L^−1^ of Asp, and the pH of the spiked samples was adjusted to the optimized condition (pH = 6). 25 mL of each spiked sample was added to 25 mg of PMCNTs and 25 mg of FMCNTs in a separate 50 mL plastic tube. The mixture was shaken continuously for 30 minutes, then filtered through a nylon syringe filter (0.45 µm). The un spiked samples were used as blank, while the spiked solutions were used as standards in order to evaluate the adsorption of Asp by PMCNT and FMCNT appropriately. A UV-VIS spectrophotometer was applied for absorbance measurement and the adsorption percentages of Asp on PMCNT and FMCNT were calculated.

## Data Availability

The datasets generated during and/or analysed during the current study are available from the corresponding author on reasonable request.

## References

[1] Gadipelly C (2014). Pharmaceutical industry wastewater: review of the technologies for water treatment and reuse. Industrial & Engineering Chemistry Research.

[2] Al-Khateeb LA, Almotiry S, Salam MA (2014). Adsorption of pharmaceutical pollutants onto graphene nanoplatelets. Chemical Engineering Journal.

[3] Shraim A (2017). Analysis of some pharmaceuticals in municipal wastewater of Almadinah Almunawarah. Arabian Journal of Chemistry.

[4] Al Qarni H, Collier P, O’Keeffe J, Akunna J (2016). Investigating the removal of some pharmaceutical compounds in hospital wastewater treatment plants operating in Saudi Arabia. Environmental Science and Pollution Research.

[5] Belgiorno V (2007). Review on endocrine disrupting-emerging compounds in urban wastewater: occurrence and removal by photocatalysis and ultrasonic irradiation for wastewater reuse. Desalination.

[6] Klavarioti M, Mantzavinos D, Kassinos D (2009). Removal of residual pharmaceuticals from aqueous systems by advanced oxidation processes. Environment international.

[7] Westerhoff P, Moon H, Minakata D, Crittenden J (2009). Oxidation of organics in retentates from reverse osmosis wastewater reuse facilities. Water research.

[8] Radjenovic J, Petrovic M, Barceló D (2007). Analysis of pharmaceuticals in wastewater and removal using a membrane bioreactor. Analytical and bioanalytical chemistry.

[9] Akkari M (2018). ZnO/sepiolite heterostructured materials for solar photocatalytic degradation of pharmaceuticals in wastewater. Applied Clay Science.

[10] Baran W, Adamek E, Jajko M, Sobczak A (2018). Removal of veterinary antibiotics from wastewater by electrocoagulation. Chemosphere.

[11] Shan D (2018). Intercalation of rigid molecules between carbon nanotubes for adsorption enhancement of typical pharmaceuticals. Chemical Engineering Journal.

[12] Ncibi MC, Sillanpää M (2017). Optimizing the removal of pharmaceutical drugs Carbamazepine and Dorzolamide from aqueous solutions using mesoporous activated carbons and multi-walled carbon nanotubes. Journal of Molecular Liquids.

[13] Yanyan L, Kurniawan TA, Albadarin AB, Walker G (2018). Enhanced removal of acetaminophen from synthetic wastewater using multi-walled carbon nanotubes (MWCNTs) chemically modified with NaOH, HNO_3_/H_2_SO_4_, ozone, and/or chitosan. Journal of Molecular Liquids.

[14] Álvarez-Torrellas S, Rodríguez A, Ovejero G, García J (2016). Comparative adsorption performance of ibuprofen and tetracycline from aqueous solution by carbonaceous materials. Chemical Engineering Journal.

[15] Ncibi MC, Sillanpää M (2015). Optimized removal of antibiotic drugs from aqueous solutions using single, double and multi-walled carbon nanotubes. Journal of hazardous materials.

[16] Zhao H (2016). Adsorption behavior and mechanism of chloramphenicols, sulfonamides, and non-antibiotic pharmaceuticals on multi-walled carbon nanotubes. Journal of hazardous materials.

[17] Azman A (2019). Effect of Adsorption Parameter on the Removal of Aspirin Using Tyre Waste Adsorbent. Chemical Engineering Transactions.

[18] Camilli L (2012). Structural, electronic and photovoltaic characterization of multiwalled carbon nanotubes grown directly on stainless steel. Beilstein journal of nanotechnology.

[19] Seo JW (2003). Synthesis and manipulation of carbon nanotubes. New Journal of Physics.

[20] Schönherr J, Buchheim J, Scholz P, Adelhelm P (2018). Boehm titration revisited (part i): Practical aspects for achieving a high precision in quantifying oxygen-containing surface groups on carbon materials. C.

[21] Acharya J, Sahu J, Mohanty C, Meikap B (2009). Removal of lead (II) from wastewater by activated carbon developed from Tamarind wood by zinc chloride activation. Chemical Engineering Journal.

[22] Rakić V, Rajić N, A. Daković A (2013). Auroux, The adsorption of salicylic acid, acetylsalicylic acid and atenolol from aqueous solutions onto natural zeolites and clays: clinoptilolite, bentonite and kaolin. Microporous and Mesoporous Materials.

[23] Yoon S-D, Byun H-S (2013). Molecularly imprinted polymers for selective separation of acetaminophen and aspirin by using supercritical fluid technology. Chemical Engineering Journal.

[24] Byun H-S, Youn Y-N, Yun Y-H, Yoon S-D (2010). Selective separation of aspirin using molecularly imprinted polymers. Separation and Purification Technology.

